# Understanding global practice patterns in bulbar urethral reconstruction to inform urethroplasty training

**DOI:** 10.1007/s00345-026-06904-1

**Published:** 2026-11-03

**Authors:** Amir A. Feinberg, Hiren V. Patel, Eric Walton, Tasha Posid, Pankaj M. Joshi, Brian M. Inouye, George E. Koch

**Affiliations:** 1 Department of Urology, The Ohio State University Wexner Medical Center, 915 Olentangy River Rd, Columbus, OH 43212 USA; 2 UROKUL Urology Institute, Pune, India; 3 Department of Urology, Albany Medical Center, Albany, NY USA

**Keywords:** Bulbar urethral stricture, Urethroplasty, Reconstructive urology, Practice patterns

## Abstract

**Purpose:**

To characterize global variation in operative decision-making for bulbar urethral strictures and identify surgeon-level factors associated with technique selection and intra-surgeon concordance.

**Methods:**

A cross-sectional survey was distributed to members of two large international reconstructive urological societies. Participants selected preferred techniques for standardized short (1 cm) and long (3–4 cm) bulbar strictures presented as written scenarios and corresponding retrograde urethrograms (RUGs). Concordance was defined as consistent technique selection across formats for the same stricture. Associations between preferences, concordance, and surgeon characteristics were analyzed.

**Results:**

A total of 167 surgeons from 38 countries responded; 40.7% completed formal reconstructive fellowship training. Technique selection for short strictures varied significantly by both training region (*p* < 0.001) and practice region (*p* < 0.001), whereas preferences for long strictures were consistent, with a majority favoring dorsal onlay buccal grafting. Years in practice and case volume were not associated with technique choice. Overall concordance was low, particularly for short strictures (33.5% vs. 50.9% for long, *p* < 0.01). Concordance was higher among U.S.-trained, fellowship-trained, and U.S.-practicing surgeons for both long and short strictures. For long strictures, concordance was 65.1% vs. 42.3% (*p* < 0.01) by training region, 66.2% vs. 40.4% (*p* < 0.01) by fellowship status, and 63.6% vs. 44.6% (*p* = 0.03) by practice region. For short strictures, concordance was 44.4% vs. 26.9% (*p* = 0.03) by training region, 48.5% vs. 23.2% (*p* < 0.01) by fellowship status, and 50.9% vs. 25.0% (*p* < 0.01) by practice region.

**Conclusions:**

Substantial global variability exists in the management of bulbar urethral strictures, especially for short strictures. Surgeon experience and volume do not predict technique selection or decision consistency, whereas training background does. Low intra-surgeon concordance suggests that even individual surgeons do not apply a consistent decision-making process to similar presentations, a gap that structured curricula, standardized imaging interpretation, and consensus-driven frameworks could directly address.

**Supplementary Information:**

The online version contains supplementary material available at https://doi.org/10.1007/s00345-026-06904-1.

## Introduction

The bulbar urethra is the most common site of urethral stricture, with urethroplasty remaining the gold standard repair, regardless of technique [1–5]. However, the choice of urethroplasty approach varies among reconstructive surgeons, and the presence of multiple accepted techniques makes the development of a unified reconstructive curriculum challenging [6]. A multi-institutional study from the Society of Genitourinary Reconstructive Surgeons (GURS) demonstrated substantial variability in the surgical management of similar strictures, both across surgeons and within individual surgeons over time. Concurrently, anterior urethroplasty techniques have continued to evolve, with increasing diversity in reconstructive approaches and graft placement strategies among reconstructive surgeons [7, 8]. This demonstrated variability may signify technical evolution over time, subtleties in surgical decision-making, or the lack of agreement on a current “best approach” to the bulbar urethra.

In clinical settings, many nuanced data points may influence surgical decision-making with regard to approach. Although prior work has assessed variability in urethroplasty practice patterns, little is known about how individual surgeons make operative decisions when presented with various strictures. It is also unclear how much surgeon-level factors, such as fellowship training, geographic background, or available resources influence that decision-making.

The objective of this study was to characterize global patterns in technique selection for bulbar urethral strictures, explore factors that may influence surgical decision-making, and evaluate surgeon-level concordance in treatment planning across descriptive and radiographic case presentations. We hypothesized that surgeons with more experience and those in international practice settings would favor anastomotic approaches. We further hypothesized that surgeons would demonstrate high intra-surgeon concordance for repair preferences when presented with bulbar urethral strictures described in written and radiographic formats. By exploring the current global practice patterns of reconstructive urologists, we hope to inform future reconstructive training curricula.

## Methods

A cross-sectional survey was administered using REDCap to members of GURS and the International Society of Reconstructive Urology (ISORU) (IRB 20250679). These organizations were selected to represent a broad international cohort of surgeons specializing in male urethral reconstruction. Eligible participants included practicing urologists who perform urethroplasty and were members of either GURS or ISORU. Participation was voluntary, and responses were collected anonymously.

The survey instrument consisted of two primary components (Supplemental Fig. 1). First, participants were presented with standardized written descriptions of non-obliterated bulbar urethral strictures differing by length and location. For this study, short strictures were defined as 1 cm in length and long strictures as 3–4 cm in length. Descriptions included length, degree of obliteration, and bulbar location. Second, respondents were shown retrograde urethrogram (RUG) images that corresponded to each of the written scenarios. The images were selected to represent typical radiographic appearances of the previously described strictures and were reviewed by expert reconstructive urologists to ensure clarity and representativeness. Neither the descriptions nor the RUG images conveyed obliterative pathology. For each scenario and RUG, surgeons selected their preferred operative technique from a standardized list of potential options (e.g., excision and primary anastomosis (EPA), non-transecting anastomotic, dorsal onlay buccal graft urethroplasty, etc.) Demographic and training-related variables included years in practice, region of reconstructive training, fellowship training in genitourinary reconstruction, and approximate annual urethroplasty volume.

Concordance of surgical approach was defined as selecting the same operative technique for a written description and all associated RUG images representing that same stricture category. Concordance was calculated separately for short (1 cm) and long (3–4 cm) bulbar strictures. Before comparison, anastomotic technique labels were harmonized across the two presentation formats: “non-transecting excision and primary anastomosis” (written scenario) and “non-transecting” (RUG) were both scored as non-transecting anastomotic, and “transecting excision and primary anastomosis” (written scenario) and “excision and primary anastomosis” (RUG) were both scored as transecting anastomotic (EPA). All other technique options were compared as selected. Free-text “Other” responses (e.g., Optilume, double-face buccal mucosal graft) were retained as “Other” and considered concordant only if selected across the written scenario and both corresponding RUGs for a given stricture length. Technique preferences and concordance rates were compared across surgeon subgroups using chi-square or Fisher’s exact tests, as appropriate. To compare concordance between the stricture groups, we used McNemar’s Test as respondents were the same for both groups. Statistical analyses were performed using Python 3.10 in Google Colab with the pandas and SciPy statistical libraries. Statistical significance was defined as p-value < 0.05.

## Results

**Fig. 1:**
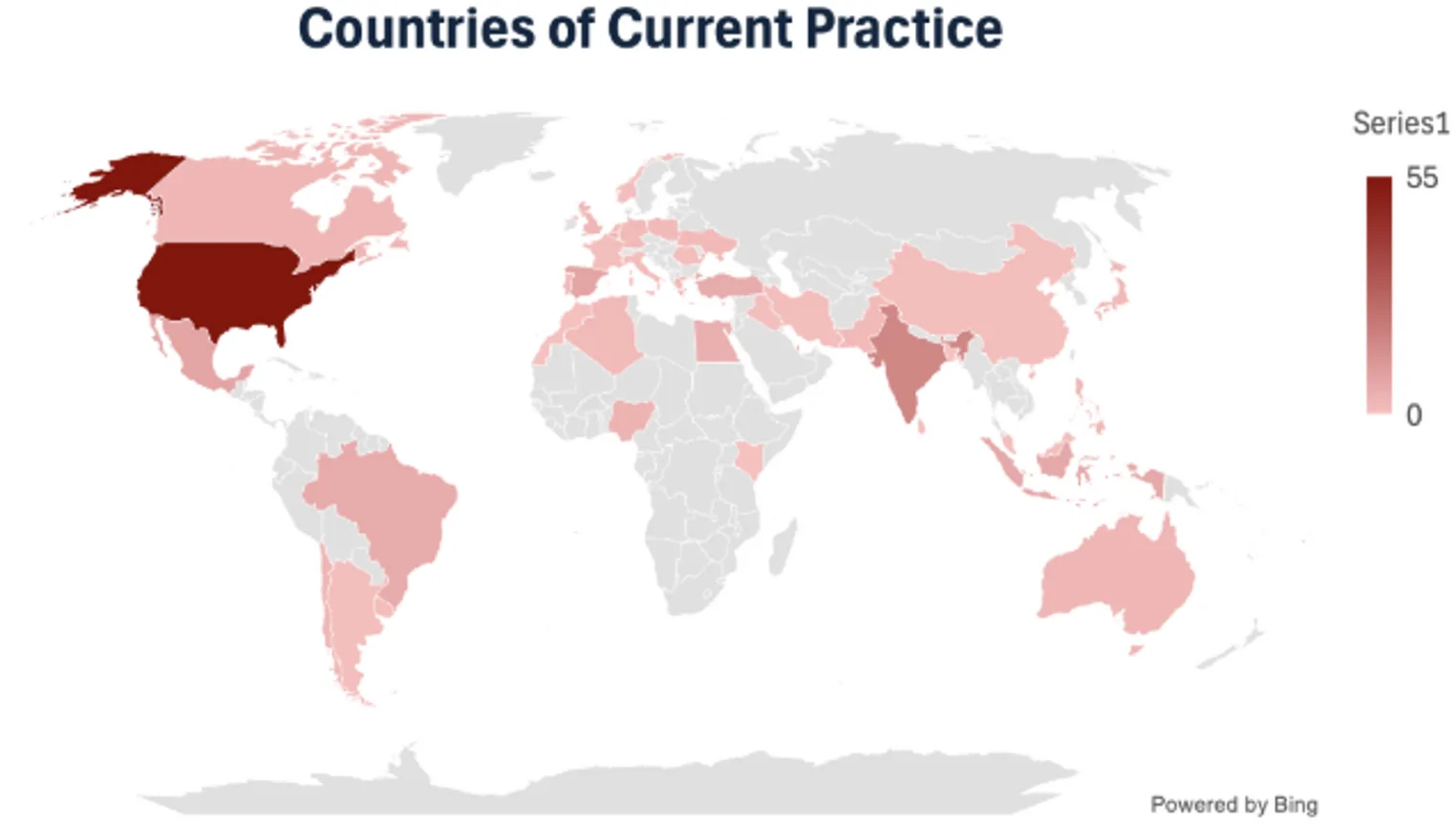
Global choropleth map illustrating countries of current practice

A total of 167 reconstructive urologists from 38 countries completed the survey and were included in the analysis, representing approximately 13.4% of the 1,250 active members across ISORU (*n* = 800) and GURS (*n* = 450), not accounting for surgeons who are active members of both societies. Respondents represented a broad international cohort, with sizable groups of both U.S.-trained and non-U.S.-trained surgeons. Only 68 (40.7%) reported completing a formal reconstructive fellowship. Complete surgeon demographics can be found in Table 1. Respondents’ countries of practice are shown in Fig. 1​.

Preferred operative approaches for short (1 cm) bulbar urethral strictures differed significantly by training region (*p* < 0.01, Table 2). In contrast, there were no significant regional differences in technique preference for long (3–4 cm) strictures (written scenario *p* = 0.37; Stricture 3 *p* = 0.68; Stricture 4 *p* = 0.26). For longer strictures, the majority of surgeons selected dorsal onlay buccal mucosal grafts. Preferences for short strictures did not significantly differ by years in practice (*p* = 0.41), annual urethroplasty volume (*p* = 0.31), or urethroplasty volume during training (*p* = 0.12). Preferences for long strictures likewise did not significantly differ by years in practice (*p* = 0.10), annual urethroplasty volume (*p* = 0.40), or urethroplasty volume during training (*p* = 0.91).

For the total cohort, intra-surgeon concordance between written descriptions and RUGs was significantly lower for short strictures (33.5%) than for long strictures (50.9%, *p* < 0.01). Years in practice and urethroplasty volume were not associated with concordance for either stricture category (Table 3). Training region, fellowship training status, and practice region were each significantly associated with concordance for both stricture lengths. U.S.-trained surgeons showed higher concordance than non-U.S.-trained surgeons (short 44.4% vs. 26.9%, *p* = 0.03, long 65.1% vs. 42.3%, *p* < 0.01). Fellowship-trained surgeons showed higher concordance than those without fellowship training (short 48.5% vs. 23.2%, *p* < 0.01, long 66.2% vs. 40.4%, *p* < 0.01). U.S.-practicing surgeons showed higher concordance than non-U.S.-practicing surgeons (short 50.9% vs. 25.0%, *p* < 0.01, long 63.6% vs. 44.6%, *p* = 0.03) (Table 3).

## Discussion

This international survey demonstrates substantial global variability in operative technique selection for bulbar urethral strictures, particularly for shorter strictures. While preferences for long strictures appeared relatively consistent across regions, U.S.-trained surgeons and internationally trained surgeons demonstrated divergent technique selection patterns for short strictures, with U.S.-trained surgeons favoring anastomotic approaches more often than non-U.S.-trained surgeons (52/63, 82.5% vs. 53/104, 51.0%). Interestingly, surgeon experience and annual case volume did not impact approach choice. Finally, repair-choice concordance for strictures of the same length and location was surprisingly low at 33.5% and 50.9% for short and long strictures, respectively. For longer strictures, surgeons most commonly selected a dorsal onlay approach based on both written case descriptions and RUG images. However, presentation of RUG imaging was associated with a shift toward more standardized graft-based reconstruction. For example, selection of dorsal onlay increased modestly (61.7% written vs. 62.3% Stricture 3 vs. 66.5% Stricture 4) and selection of augmented anastomotic technique rose more markedly (2.4% vs. 4.8% vs. 9.6%), while selection of ventral onlay actually declined (28.7% vs. 24.0% vs. 14.4%). This inconsistency, both across surgeons and within individual surgeons’ own decisions, confirms that repair choice in bulbar urethroplasty depends on more than a single objective algorithm [7, 9]. This has implications for both practical and didactic reconstructive educational initiatives, as it signals the need for instruction on multiple surgical approaches to the bulbar urethra, as well as an educational discourse of the potential influences that weigh into the decision to choose one repair over another. Finally, the associations between both approach variability and concordance with surgeon educational experience, and lack of associations with volume or operative experience, underscore the strong influence of training on a surgeon’s ultimate practice patterns.

The present study provides novel insight into intra-surgeon consistency in operative decision-making when information is presented in different formats. Surgeons demonstrated greater concordance for long strictures, which may reflect the relative clarity of indication for substitution in longer strictures, and the evidence to support oral graft material, regardless of whether the decision is based on written or radiographic information [2]. By contrast, short strictures exhibited notably lower concordance. This may be due to the inherently nuanced decision-making required for short strictures, where factors such as the quality of lumen and spongiosum, degree of fibrosis, exact location of the stricture, training background and exposure, and surgeon philosophy regarding spongiosal transection may weigh more heavily and may not be fully discernible by either text or RUG image alone [10, 11].

Training-related factors appear to be associated with repair choice concordance for both stricture lengths. U.S.-trained, fellowship-trained, and U.S.-practicing surgeons each demonstrated significantly higher concordance than their counterparts for both short and long strictures. This may reflect differences in reconstructive training environments, including differences in available resources, surgical volume, imaging exposure, cultural practice patterns, and dissemination of operative approaches across geographic regions and fellowship programs. Additionally, some degree of concordance may reflect adoption of mentor-specific operative preferences during training, resulting in persistence of established reconstructive patterns across subsequent practice [12]. Interestingly, our data did not demonstrate differences in repair choice or concordance when stratified by surgeon experience and volume. Previously, Faris et al. reported that the learning curve for urethroplasty achieves proficiency (> 90% success) after approximately 100 cases for all types of urethral reconstruction and 70 cases specifically for bulbar urethroplasty [13]. Success rates improve significantly with surgeon experience, and complications decrease over time [13, 14]. Together, these findings point to initial training, rather than years in practice or case volume, as the main driver of how surgeons approach these strictures. A standardized urethroplasty curriculum could use our data on repair variability and decision-making to shape its content, though case volume will still matter for building technical proficiency. Giving trainees an explicit framework for approach selection, rather than letting it develop unevenly through individual mentorship, could produce more consistent decision-making earlier in training.

This study has several limitations. While the goal of the study was to improve understanding of surgeon preference and decision-making, these are inherently difficult qualities to quantify and may ultimately be better suited to qualitative research. RUG images may not fully capture the degree of fibrosis or the contours of the spongiosum, which are key determinants in operative planning. Additionally, incidental radiographic features in the RUG images beyond the intended stricture length and location may have independently influenced technique selection, representing a potential confounder in our concordance analysis. Surgeon selection was dependent on GURS and ISORU membership and may not reflect all practicing reconstructive urologists worldwide. It is also important to consider potential selection bias among survey respondents, as well as the relatively low overall response rate. However, it should also be noted that many surgeons are members of both GURS and ISORU, which potentially underestimates our calculated response rate. Finally, stated preferences may not always align with intraoperative decisions in real clinical scenarios. Despite these limitations, the findings provide meaningful insight into global variability in urethroplasty technique selection and highlight opportunities for enhanced educational standardization.

## Conclusion

In this international survey of bulbar urethral surgeons, country of training was associated with bulbar urethral stricture repair choices, but surgeon experience and volume were not. Intra-surgeon repair choice concordance was surprisingly low. Training programs aiming to reduce this variability should prioritize decision-making frameworks and structured imaging interpretation over simply increasing case volume. As reconstructive urology continues to globalize, these findings offer a concrete starting point for harmonizing how bulbar urethroplasty is taught across training environments.

**Table 1:** Demographic, Training, and Practice Characteristics of Survey Respondents

Characteristic	Total (*N* = 167)	U.S.-Trained (*N* = 63)	Non–U.S.-Trained (*n* = 104)	*P*-value
Region of current practice, *n* (%)				
*United States* *Outside of the United States*	55 (32.9%) 112 (67.1%)	54 (85.7%) 9 (14.3%)	1 (1.0%) 103 (99.0%)	**< 0.01**
Formal reconstructive fellowship completed, n (%)	68 (40.7%)	49 (77.8%)	19 (18.3%)	**< 0.01**
Years in independent reconstructive practice, n (%)				
≤5 years *5–15 years* *≥16 years*	53 (31.7%) 81 (48.5%) 33 (19.8%)	20 (31.7%) 29 (46.0%) 14 (22.2%)	33 (31.7%) 52 (50.0%) 19 (18.3%)	0.80
Anterior urethroplasties performed during training, n (%)				
*≤60 cases* *61–75 cases* *≥76 cases*	85 (50.9%) 37 (22.2%) 45 (26.9%)	17 (27.0%) 18 (28.6%) 28 (44.4%)	68 (65.4%) 19 (18.3%) 17 (16.3%)	**< 0.01**
Anterior urethroplasties performed in the past year, n (%)				
*≤20 cases* *21–40 cases* *41–60 cases* *≥61 cases*	62 (37.1%) 59 (35.3%) 21 (12.6%) 25 (15.0%)	22 (34.9%) 20 (31.7%) 9 (14.3%) 12 (19.0%)	40 (38.5%) 39 (37.5%) 12 (11.5%) 13 (12.5%)	0.60
Primary Practice Setting, n (%)				
*Academic* *Private* *Other*	113 (67.7%) 47 (28.1%) 7 (4.2%)	50 (79.4%) 10 (15.9%) 3 (4.8%)	63 (60.6%) 37 (35.6%) 4 (3.8%)	**0.02**
Works with trainees (residents or fellows), n (%)	136 (81.4%)	57 (90.5%)	79 (76.0%)	**0.03**

**Table 2:** Urethroplasty Approaches Based on Training Location

Characteristic	U.S. (*N* = 63)	Non–U.S. (*n* = 104)	*P*-value
“1 cm proximal, non-obliterative bulbar urethral stricture”			
*Augmented Anastomotic* *Dorsal Buccal Inlay* *Dorsal Buccal Onlay* *Non-Transecting Anastomotic* *Transecting Anastomotic* *Ventral Buccal Onlay* *Other*	0 (0.0%) 1 (1.6%) 7 (11.1%) 29 (46.0%) 23 (36.5%) 2 (3.2%) 1 (1.6%)	1 (1.0%) 0 (0.0%) 20 (19.2%) 43 (41.3%) 10 (9.6%) 28 (26.9%) 2 (1.9%)	**< 0.01**
“3–4 cm proximal, non-obliterative bulbar urethral stricture”			
*Augmented Anastomotic* *Dorsal Buccal Inlay* *Dorsal Buccal Onlay* *Non-Transecting Anastomotic* *Transecting Anastomotic* *Ventral Buccal Onlay* *Other*	3 (4.8%) 2 (3.2%) 38 (60.3%) 0 (0.0%) 1 (1.6%) 17 (27.0%) 2 (3.2%)	1 (1.0%) 4 (3.8%) 65 (62.5%) 1 (1.0%) 2 (1.9%) 31 (29.8%) 0 (0.0%)	0.37
Stricture 1			
*Augmented Anastomotic* *Dorsal Buccal Inlay* *Dorsal Buccal Onlay* *Non-Transecting Anastomotic* *Transecting Anastomotic* *Ventral Buccal Onlay* *Other*	1 (1.6%) 1 (1.6%) 5 (7.9%) 33 (52.4%) 18 (28.6%) 3 (4.8%) 2 (3.2%)	3 (2.9%) 1 (1.0%) 18 (17.3%) 54 (51.9%) 9 (8.7%) 18 (17.3%) 1 (1.0%)	**< 0.01**
Stricture 2			
*Augmented Anastomotic* *Dorsal Buccal Inlay* *Dorsal Buccal Onlay* *Non-Transecting Anastomotic* *Transecting Anastomotic* *Ventral Buccal Onlay* *Skin Flap Augmentation* *Other*	3 (4.8%) 0 (0.0%) 8 (12.7%) 15 (23.8%) 32 (50.8%) 4 (6.3%) 0 (0.0%) 1 (1.6%)	4 (3.8%) 1 (1.0%) 23 (22.1%) 19 (18.3%) 26 (25.0%) 24 (23.1%) 1 (1.0%) 6 (5.8%)	**< 0.01**
Stricture 3			
*Augmented Anastomotic* *Dorsal Buccal Inlay* *Dorsal Buccal Onlay* *Non-Transecting Anastomotic* *Transecting Anastomotic* *Ventral Buccal Onlay* *Skin Flap Augmentation* *Other*	5 (7.9%) 2 (3.2%) 39 (61.9%) 1 (1.6%) 1 (1.6%) 14 (22.2%) 0 (0.0%) 1 (1.6%)	3 (2.9%) 4 (3.8%) 65 (62.5%) 0 (0.0%) 2 (1.9%) 26 (25.0%) 1 (1.0%) 3 (2.9%)	0.68
Stricture 4			
*Augmented Anastomotic* *Dorsal Buccal Inlay* *Dorsal Buccal Onlay* *Ventral Buccal Onlay* *Skin Flap Augmentation* *Perineal Urethrostomy* *Other*	10 (15.9%) 3 (4.8%) 38 (60.3%) 9 (14.3%) 0 (0.0%) 1 (1.6%) 2 (3.2%)	6 (5.8%) 4 (3.8%) 73 (70.2%) 15 (14.4%) 2 (1.9%) 0 (0.0%) 4 (3.8%)	0.26

**Table 3:** Associations with Intra-surgeon Concordance

Characteristic	Short Stricture Concordance	*P*-value	Long Stricture Concordance	*P*-value
Region of Reconstructive Practice, %				
*United States* *Outside of the United States*	28/55 (50.9%) 28/112 (25.0%)	**< 0.01**	35/55 (63.6%) 50/112 (44.6%)	**0.03**
Region of Reconstructive Training, %				
*United States* *Outside of the United States*	28/63 (44.4%) 28/104 (26.9%)	**0.03**	41/63 (65.1%) 44/104 (42.3%)	**< 0.01**
Formal reconstructive fellowship completed, %				
*Yes* *No*	33/68 (48.5%) 23/99 (23.2%)	**< 0.01**	45/68 (66.2%) 40/99 (40.4%)	**< 0.01**
Years in independent reconstructive practice, %				
*≤5 years* *5–15 years* *≥16 years*	17/53 (32.1%) 28/81 (34.6%) 11/33 (33.3%)	0.96	32/53 (60.4%) 38/81 (46.9%) 15/33 (45.5%)	0.24
Anterior urethroplasties performed during training, %				
*≤60 cases* *61–75 cases* *≥76 cases*	22/85 (25.9%) 16/37 (43.2%) 18/45 (40.0%)	0.10	38/85 (44.7%) 19/37 (51.4%) 28/45 (62.2%)	0.16
Anterior urethroplasties performed in the past year, %				
*≤20 cases* *21–40 cases* *41–60 cases* *≥61 cases*	16/62 (25.8%) 19/59 (32.2%) 10/21 (47.6%) 11/25 (44.0%)	0.19	33/62 (53.2%) 29/59 (49.2%) 10/21 (47.6%) 13/25 (52.0%)	0.96

## Supplementary Information

Below is the link to the electronic supplementary material.

Supplementary Material 1

## Data Availability

The data supporting the findings of this study are available within the manuscript and its supplementary materials. Additional data collected during the study are stored in a secure REDCap database and are not publicly available due to privacy restrictions, but may be made available to qualified researchers upon reasonable request to the corresponding author.
