# Supplementary material for: Understanding global practice patterns in bulbar urethral reconstruction to inform urethroplasty training
Source: World J Urol. 2026 Nov 3;44(1):782. doi: 10.1007/s00345-026-06904-1 (PMC13731513; doi:10.1007/s00345-026-06904-1)
Supplement: Supplementary file 1 [file 345_2026_6904_MOESM1_ESM.pdf]

Urethroplasty Survey

Please complete this brief survey on bulbar urethroplasty reconstructive preferences to help us understand what current practice patterns, training, and preferences look like in the reconstructive community. Your answers will be anonymous. By completing the survey, you consent to your anonymous answers being included in our final dataset.

Thank you so much for your time.

In what country do you currently practice?

- ◯ Afghanistan
- ◯ Albania
- ◯ Algeria
- ◯ Andorra
- ◯ Angola
- ◯ Antigua and Barbuda
- ◯ Argentina
- ◯ Armenia
- ◯ Australia
- ◯ Austria
- ◯ Azerbaijan
- ◯ Bahamas
- ◯ Bahrain
- ◯ Bangladesh
- ◯ Barbados
- ◯ Belarus
- ◯ Belgium
- ◯ Belize
- ◯ Benin
- ◯ Bhutan
- ◯ Bolivia
- ◯ Bosnia and Herzegovina
- ◯ Botswana
- ◯ Brazil
- ◯ Brunei
- ◯ Bulgaria
- ◯ Burkina Faso
- ◯ Burundi
- ◯ Cabo Verde
- ◯ Cambodia
- ◯ Cameroon
- ◯ Canada
- ◯ Central African Republic
- ◯ Chad
- ◯ Chile
- ◯ China
- ◯ Colombia
- ◯ Comoros
- ◯ Congo (Congo-Brazzaville)
- ◯ Costa Rica
- ◯ Croatia
- ◯ Cuba
- ◯ Cyprus
- ◯ Czech Republic (Czechia)
- ◯ Democratic Republic of the Congo (Congo-Kinshasa)
- ◯ Denmark
- ◯ Djibouti
- ◯ Dominica
- ◯ Dominican Republic
- ◯ East Timor (Timor-Leste)
- ◯ Ecuador
- ◯ Egypt
- ◯ El Salvador
- ◯ Equatorial Guinea
- ◯ Eritrea
- ◯ Estonia
- ◯ Eswatini (Swaziland)
- ◯ Ethiopia
- ◯ Fiji
- ◯ Finland
- ◯ France
- ◯ Gabon
- ◯ Gambia
- ◯ Georgia
- ◯ Germany
- ◯ Ghana
- ◯ Greece
- ◯ Grenada
- ◯ Guatemala

◯ Guinea
◯ Guinea-Bissau
◯ Guyana
◯ Haiti
◯ Honduras
◯ Hungary
◯ Iceland
◯ India
◯ Indonesia
◯ Iran
◯ Iraq
◯ Ireland
◯ Israel
◯ Italy
◯ Ivory Coast (Côte d'Ivoire)
◯ Jamaica
◯ Japan
◯ Jordan
◯ Kazakhstan
◯ Kenya
◯ Kiribati
◯ Korea, North (North Korea)
◯ Korea, South (South Korea)
◯ Kuwait
◯ Kyrgyzstan
◯ Laos
◯ Latvia
◯ Lebanon
◯ Lesotho
◯ Liberia
◯ Libya
◯ Liechtenstein
◯ Lithuania
◯ Luxembourg
◯ Madagascar
◯ Malawi
◯ Malaysia
◯ Maldives
◯ Mali
◯ Malta
◯ Marshall Islands
◯ Mauritania
◯ Mauritius
◯ Mexico
◯ Micronesia
◯ Moldova
◯ Monaco
◯ Mongolia
◯ Montenegro
◯ Morocco
◯ Mozambique
◯ Myanmar (Burma)
◯ Namibia
◯ Nauru
◯ Nepal
◯ Netherlands
◯ New Zealand
◯ Nicaragua
◯ Niger
◯ Nigeria
◯ North Macedonia
◯ Norway
◯ Oman
◯ Pakistan
◯ Palau
◯ Palestine
◯ Panama
◯ Papua New Guinea
◯ Paraguay
◯ Peru
◯ Philippines

◯ Poland
◯ Portugal
◯ Qatar
◯ Romania
◯ Russia
◯ Rwanda
◯ Saint Kitts and Nevis
◯ Saint Lucia
◯ Saint Vincent and the Grenadines
◯ Samoa
◯ San Marino
◯ Sao Tome and Principe
◯ Saudi Arabia
◯ Senegal
◯ Serbia
◯ Seychelles
◯ Sierra Leone
◯ Singapore
◯ Slovakia
◯ Slovenia
◯ Solomon Islands
◯ Somalia
◯ South Africa
◯ South Sudan
◯ Spain
◯ Sri Lanka
◯ Sudan
◯ Suriname
◯ Sweden
◯ Switzerland
◯ Syria
◯ Tajikistan
◯ Tanzania
◯ Thailand
◯ Togo
◯ Tonga
◯ Trinidad and Tobago
◯ Tunisia
◯ Turkey
◯ Turkmenistan
◯ Tuvalu
◯ Uganda
◯ Ukraine
◯ United Arab Emirates
◯ United Kingdom
◯ United States of America
◯ Uruguay
◯ Uzbekistan
◯ Vanuatu
◯ Vatican City (Holy See)
◯ Venezuela
◯ Vietnam
◯ Yemen
◯ Zambia
◯ Zimbabwe

In what country did you train to do urologic reconstruction?<br>(E.g. fellowship or post-residency training specifically in reconstruction)	◯ Afghanistan<br>◯ Albania<br>◯ Algeria<br>◯ Andorra<br>◯ Angola<br>◯ Antigua and Barbuda<br>◯ Argentina<br>◯ Armenia<br>◯ Australia<br>◯ Austria<br>◯ Azerbaijan<br>◯ Bahamas<br>◯ Bahrain<br>◯ Bangladesh<br>◯ Barbados<br>◯ Belarus<br>◯ Belgium<br>◯ Belize<br>◯ Benin<br>◯ Bhutan<br>◯ Bolivia<br>◯ Bosnia and Herzegovina<br>◯ Botswana<br>◯ Brazil<br>◯ Brunei<br>◯ Bulgaria<br>◯ Burkina Faso<br>◯ Burundi<br>◯ Cabo Verde<br>◯ Cambodia<br>◯ Cameroon<br>◯ Canada<br>◯ Central African Republic<br>◯ Chad<br>◯ Chile<br>◯ China<br>◯ Colombia<br>◯ Comoros<br>◯ Congo (Congo-Brazzaville)<br>◯ Costa Rica<br>◯ Croatia<br>◯ Cuba<br>◯ Cyprus<br>◯ Czech Republic (Czechia)<br>◯ Democratic Republic of the Congo (Congo-Kinshasa)<br>◯ Denmark<br>◯ Djibouti<br>◯ Dominica<br>◯ Dominican Republic<br>◯ East Timor (Timor-Leste)<br>◯ Ecuador<br>◯ Egypt<br>◯ El Salvador<br>◯ Equatorial Guinea<br>◯ Eritrea<br>◯ Estonia<br>◯ Eswatini (Swaziland)<br>◯ Ethiopia<br>◯ Fiji<br>◯ Finland<br>◯ France<br>◯ Gabon<br>◯ Gambia<br>◯ Georgia<br>◯ Germany<br>◯ Ghana<br>◯ Greece<br>◯ Grenada<br>◯ Guatemala

◯ Guinea
◯ Guinea-Bissau
◯ Guyana
◯ Haiti
◯ Honduras
◯ Hungary
◯ Iceland
◯ India
◯ Indonesia
◯ Iran
◯ Iraq
◯ Ireland
◯ Israel
◯ Italy
◯ Ivory Coast (Côte d'Ivoire)
◯ Jamaica
◯ Japan
◯ Jordan
◯ Kazakhstan
◯ Kenya
◯ Kiribati
◯ Korea, North (North Korea)
◯ Korea, South (South Korea)
◯ Kuwait
◯ Kyrgyzstan
◯ Laos
◯ Latvia
◯ Lebanon
◯ Lesotho
◯ Liberia
◯ Libya
◯ Liechtenstein
◯ Lithuania
◯ Luxembourg
◯ Madagascar
◯ Malawi
◯ Malaysia
◯ Maldives
◯ Mali
◯ Malta
◯ Marshall Islands
◯ Mauritania
◯ Mauritius
◯ Mexico
◯ Micronesia
◯ Moldova
◯ Monaco
◯ Mongolia
◯ Montenegro
◯ Morocco
◯ Mozambique
◯ Myanmar (Burma)
◯ Namibia
◯ Nauru
◯ Nepal
◯ Netherlands
◯ New Zealand
◯ Nicaragua
◯ Niger
◯ Nigeria
◯ North Macedonia
◯ Norway
◯ Oman
◯ Pakistan
◯ Palau
◯ Palestine
◯ Panama
◯ Papua New Guinea
◯ Paraguay
◯ Peru
◯ Philippines

◯ Poland
◯ Portugal
◯ Qatar
◯ Romania
◯ Russia
◯ Rwanda
◯ Saint Kitts and Nevis
◯ Saint Lucia
◯ Saint Vincent and the Grenadines
◯ Samoa
◯ San Marino
◯ Sao Tome and Principe
◯ Saudi Arabia
◯ Senegal
◯ Serbia
◯ Seychelles
◯ Sierra Leone
◯ Singapore
◯ Slovakia
◯ Slovenia
◯ Solomon Islands
◯ Somalia
◯ South Africa
◯ South Sudan
◯ Spain
◯ Sri Lanka
◯ Sudan
◯ Suriname
◯ Sweden
◯ Switzerland
◯ Syria
◯ Tajikistan
◯ Tanzania
◯ Thailand
◯ Togo
◯ Tonga
◯ Trinidad and Tobago
◯ Tunisia
◯ Turkey
◯ Turkmenistan
◯ Tuvalu
◯ Uganda
◯ Ukraine
◯ United Arab Emirates
◯ United Kingdom
◯ United States of America
◯ Uruguay
◯ Uzbekistan
◯ Vanuatu
◯ Vatican City (Holy See)
◯ Venezuela
◯ Vietnam
◯ Yemen
◯ Zambia
◯ Zimbabwe

How many years did you train specifically in urologic reconstruction?
(E.g. fellowship or post-residency training specifically in reconstruction)

____

Did you complete a formal fellowship in urologic reconstruction?
(E.g. a training program sanctioned by a governing body like GURS or SIU)

◯ Yes
◯ No

What specific fellowship did you complete?	◯ GURS<br>◯ FPMRS<br>◯ Other
How many years have you been in independent practice doing urologic reconstruction?	◯ ≤5 years<br>◯ 5-15 years<br>◯ ≥16 years
How many anterior urethroplasties did you do during fellowship/reconstructive training?	◯ ≤60<br>◯ 61-75<br>◯ ≥76
How many anterior urethroplasties did you do last year?	◯ ≤20<br>◯ 21-40<br>◯ 41-60<br>◯ ≥61
What is your primary practice setting?	◯ Academic<br>◯ Private<br>◯ Other
Do you work with trainees (residents or fellows)?	◯ Yes<br>◯ No
What was your fellowship director's preferred reconstructive approach to 1 cm proximal, non-obliterative bulbar urethral strictures?	◯ Transecting excision and primary anastomosis<br>◯ Non-transecting excision and primary anastomosis<br>◯ Augmented anastomotic<br>◯ Ventral buccal onlay<br>◯ Dorsal buccal onlay<br>◯ Dorsal buccal inlay<br>◯ Skin flap augmentation<br>◯ Perineal urethrostomy<br>◯ Other
Other Approach	__________________________________
In YOUR PRACTICE, what is your preferred reconstructive approach to 1 cm proximal, non-obliterative bulbar urethral strictures?	◯ Transecting excision and primary anastomosis<br>◯ Non-transecting excision and primary anastomosis<br>◯ Augmented anastomotic<br>◯ Ventral buccal onlay<br>◯ Dorsal buccal onlay<br>◯ Dorsal buccal inlay<br>◯ Skin flap augmentation<br>◯ Perineal urethrostomy<br>◯ Other
Other Approach	__________________________________

What was your fellowship director's preferred reconstructive approach to 3-4 cm proximal, non-obliterative bulbar urethral strictures?	◯ Transecting excision and primary anastomosis<br>◯ Non-transecting excision and primary anastomosis<br>◯ Augmented anastomotic<br>◯ Ventral buccal onlay<br>◯ Dorsal buccal onlay<br>◯ Dorsal buccal inlay<br>◯ Skin flap augmentation<br>◯ Perineal urethrostomy<br>◯ Other
Other Approach	__________________________________
In YOUR PRACTICE, what is your preferred reconstructive approach to 3-4 cm proximal, non-obliterative bulbar urethral strictures?	◯ Transecting excision and primary anastomosis<br>◯ Non-transecting excision and primary anastomosis<br>◯ Augmented anastomotic<br>◯ Ventral buccal onlay<br>◯ Dorsal buccal onlay<br>◯ Dorsal buccal inlay<br>◯ Skin flap augmentation<br>◯ Perineal urethrostomy<br>◯ Other
Other Approach	__________________________________

Stricture 1
Please answer the following questions for the Retrograde Urethrogram shown below:
This patient is non-irradiated, has had two prior urethrotomies and wants reconstruction.

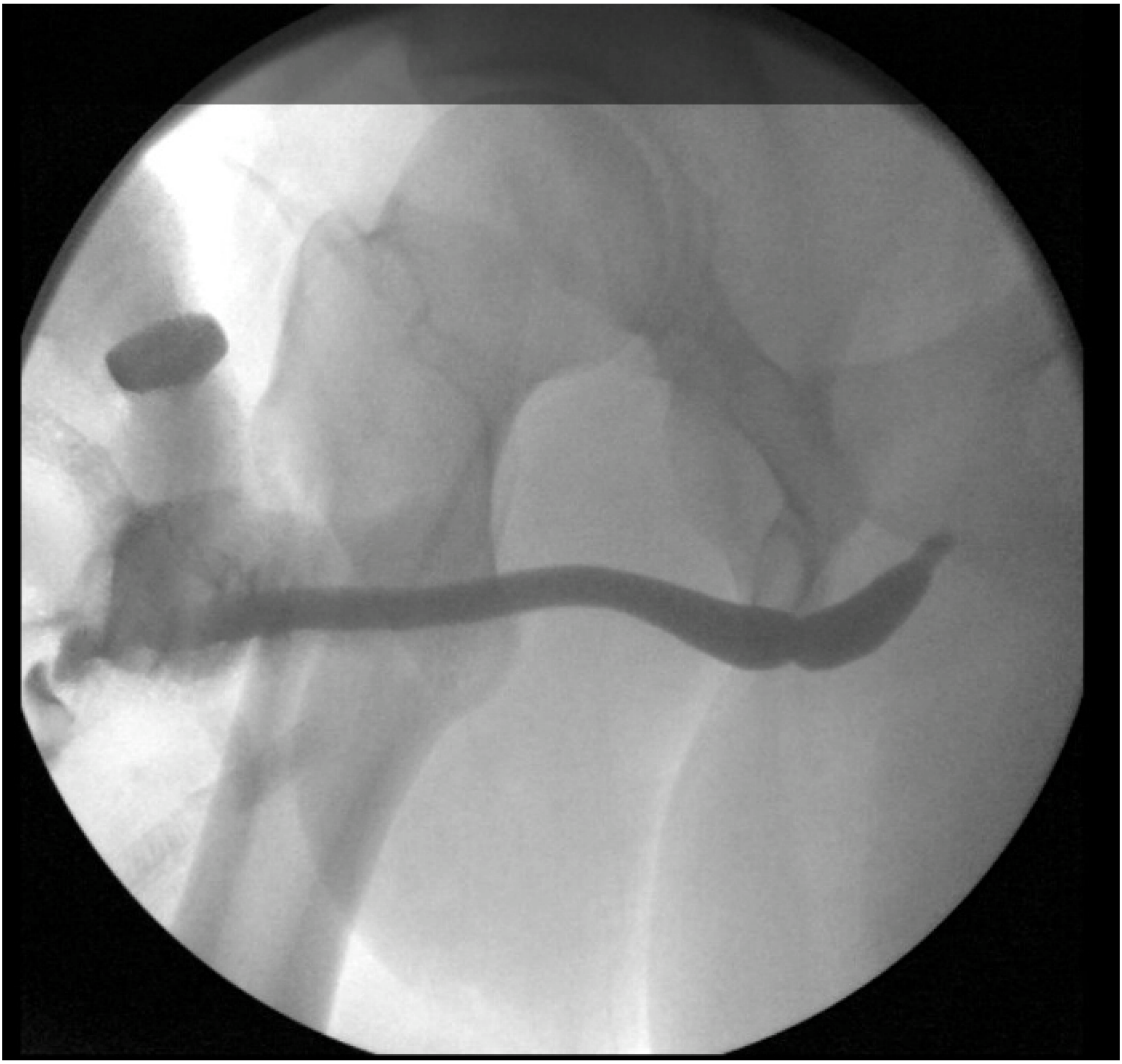

How would you reconstruct Stricture 1?

- ◯ Excision and primary anastomosis
- ◯ Non-transecting
- ◯ Augmented anastomotic
- ◯ Ventral buccal onlay
- ◯ Dorsal buccal onlay
- ◯ Dorsal buccal inlay
- ◯ Skin flap augmentation
- ◯ Perineal urethrostomy
- ◯ Other

Other Approach

____

What would you estimate is the success rate of this approach?	◯ ≤70%<br>◯ 71-80%<br>◯ 81-90%<br>◯ >90%
When would you remove the catheter following this approach?	◯ ≤7 days<br>◯ 8-14 days<br>◯ 15-21 days<br>◯ 22-28 days<br>◯ ≥29 days
Do you routinely utilize fluoroscopic imaging at the time of catheter removal following this approach?	◯ Yes<br>◯ No

Stricture 2
Please answer the following questions for the Retrograde Urethrogram shown below:
This patient is non-irradiated, has had two prior urethrotomies and wants reconstruction.

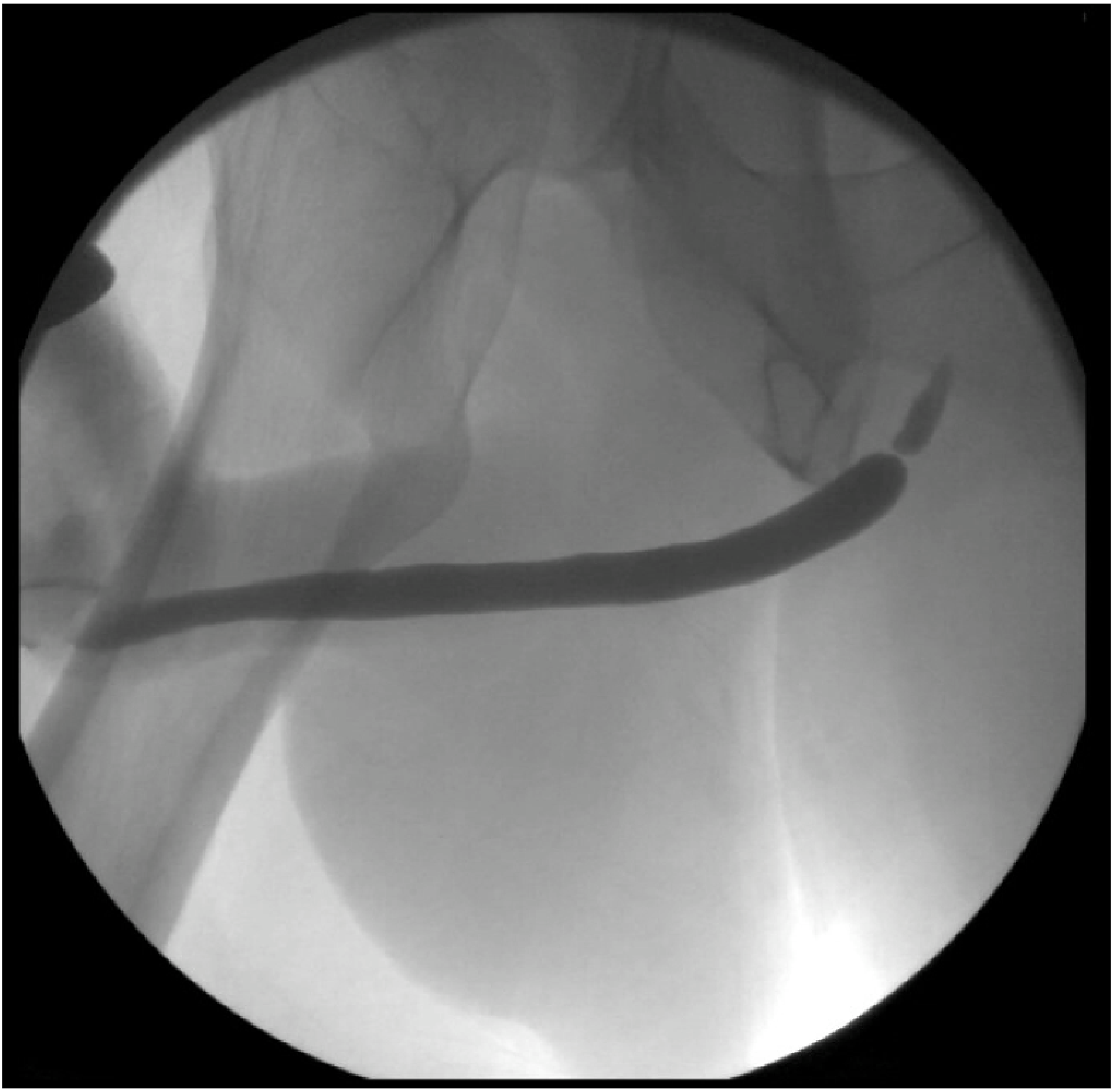

How would you reconstruct Stricture 2?	◯ Excision and primary anastomosis<br>◯ Non-transecting<br>◯ Augmented anastomotic<br>◯ Ventral buccal onlay<br>◯ Dorsal buccal onlay<br>◯ Dorsal buccal inlay<br>◯ Skin flap augmentation<br>◯ Perineal urethrostomy<br>◯ Other
Other Approach	__________________________________
What would you estimate is the success rate of this approach?	◯ ≤70%<br>◯ 71-80%<br>◯ 81-90%<br>◯ >90%
When would you remove the catheter following this approach?	◯ ≤7 days<br>◯ 8-14 days<br>◯ 15-21 days<br>◯ 22-28 days<br>◯ ≥29 days
Do you routinely utilize fluoroscopic imaging at the time of catheter removal following this approach?	◯ Yes<br>◯ No

Stricture 3
Please answer the following questions for the Retrograde Urethrogram shown below:
This patient is non-irradiated, has had two prior urethrotomies and wants reconstruction.

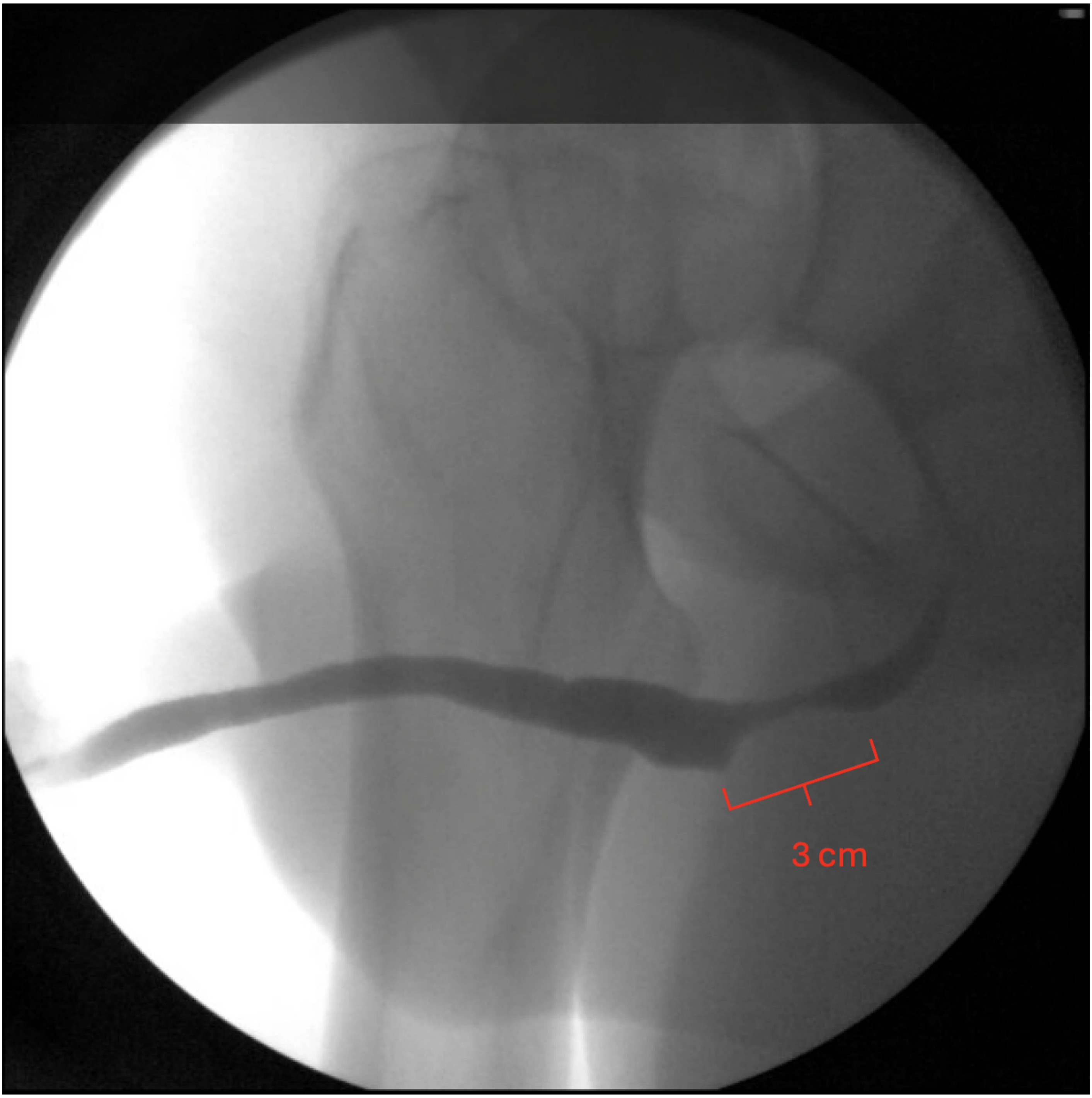

How would you reconstruct Stricture 3?

- ◯ Excision and primary anastomosis
- ◯ Non-transecting
- ◯ Augmented anastomotic
- ◯ Ventral buccal onlay
- ◯ Dorsal buccal onlay
- ◯ Dorsal buccal inlay
- ◯ Skin flap augmentation
- ◯ Perineal urethrostomy
- ◯ Other

Other Approach

____

What would you estimate is the success rate of this approach?	◯ ≤70%<br>◯ 71-80%<br>◯ 81-90%<br>◯ >90%
When would you remove the catheter following this approach?	◯ ≤7 days<br>◯ 8-14 days<br>◯ 15-21 days<br>◯ 22-28 days<br>◯ ≥29 days
Do you routinely utilize fluoroscopic imaging at the time of catheter removal following this approach?	◯ Yes<br>◯ No

Stricture 4
Please answer the following questions for the Retrograde Urethrogram shown below:
This patient is non-irradiated, has had two prior urethrotomies and wants reconstruction.

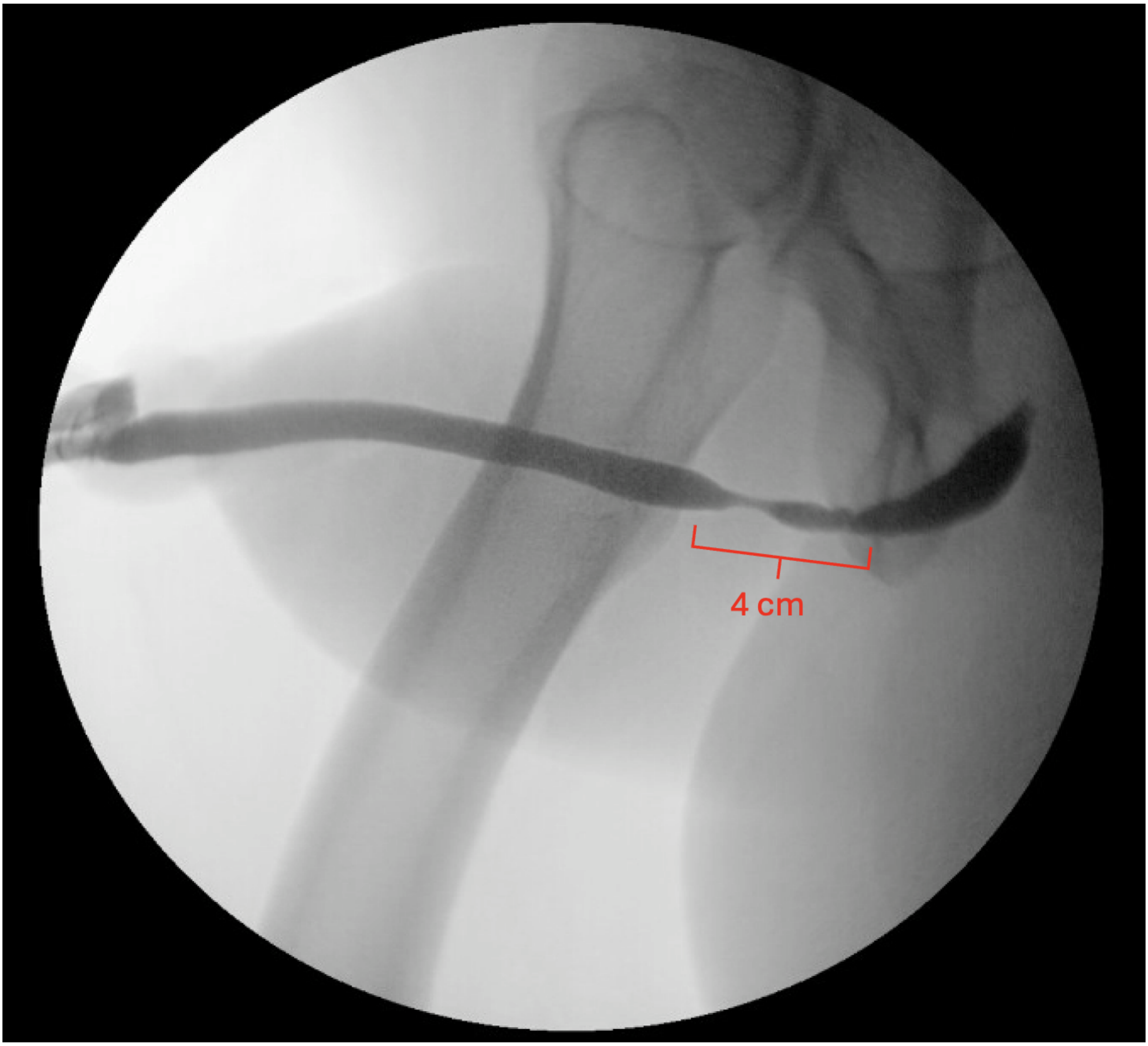

How would you reconstruct Stricture 4?	◯ Excision and primary anastomosis<br>◯ Non-transecting<br>◯ Augmented anastomotic<br>◯ Ventral buccal onlay<br>◯ Dorsal buccal onlay<br>◯ Dorsal buccal inlay<br>◯ Skin flap augmentation<br>◯ Perineal urethrostomy<br>◯ Other
Other Approach	________________________________
What would you estimate is the success rate of this approach?	◯ ≤70%<br>◯ 71-80%<br>◯ 81-90%<br>◯ >90%
When would you remove the catheter following this approach?	◯ ≤7 days<br>◯ 8-14 days<br>◯ 15-21 days<br>◯ 22-28 days<br>◯ ≥29 days
Do you routinely utilize fluoroscopic imaging at the time of catheter removal following this approach?	◯ Yes<br>◯ No
How do you define success in follow-up following bulbar urethral reconstruction?	☐ Patient-reported outcomes<br>☐ Uroflow parameters<br>☐ Post-void residual<br>☐ Passage of flexible cystoscope<br>☐ Other (specify)
Other Outcome	________________________________
At what point do you measure patient-reported outcomes?	☐ 3 months<br>☐ 6 months<br>☐ 9 months<br>☐ 12 months<br>☐ 18 months<br>☐ 24 months
At what point do you measure uroflow parameters?	☐ 3 months<br>☐ 6 months<br>☐ 9 months<br>☐ 12 months<br>☐ 18 months<br>☐ 24 months
At what point do you measure post-void residuals?	☐ 3 months<br>☐ 6 months<br>☐ 9 months<br>☐ 12 months<br>☐ 18 months<br>☐ 24 months

At what point do you perform flexible cystoscopy?

- ☐ 3 months
- ☐ 6 months
- ☐ 9 months
- ☐ 12 months
- ☐ 18 months
- ☐ 24 months

At what point do you measure "Other"?

- ☐ 3 months
- ☐ 6 months
- ☐ 9 months
- ☐ 12 months
- ☐ 18 months
- ☐ 24 months
